# Fabrication and characterization of carbon-backed thin ^208^Pb targets

**DOI:** 10.1016/j.mex.2016.10.002

**Published:** 2016-10-19

**Authors:** Meenu Thakur, R. Dubey, S.R Abhilash, B.R. Behera, B.P. Mohanty, D. Kabiraj, Sunil Ojha, Heena Duggal

**Affiliations:** a Department of Physics, Panjab University, Chandigarh – 160014, India; b Inter University Accelerator Centre, Aruna Asaf Ali Marg, New Delhi – 110067, India

**Keywords:** Vacuum evaporation method (Physical vapor deposition method), ^208^Pb, BaCl_2_, Resistive heating method, Carbon-backed

## Abstract

Thin carbon-backed isotopically enriched ^208^Pb targets were required for our experiment aimed to study the reaction dynamics for ^48^Ti + ^208^Pb system, populating the near super-heavy nucleus ^256^Rf, through mass-energy correlation of the fission fragments. Purity and thickness of the targets are of utmost importance in such studies as these factors have strong influence on the measurement accuracy of mass and energy distribution of fission fragments. ^208^Pb targets with thickness ranging from 60 μg/cm^2^ to 250 μg/cm^2^ have been fabricated in high vacuum environment using physical vapor deposition method. Important points in the method are as follows:

• ^208^Pb was deposited using resistive heating method, whereas carbon (backing foil) deposition was performed by using the electron beam bombardment technique.
• Different characterization techniques such as Particle Induced X-ray Emission (PIXE), Energy Dispersive X-Ray Fluorescence (EDXRF) and Rutherford Backscattering Spectrometry (RBS) were used to assert the purity and thickness of the targets.
• These targets have successfully been used to accomplish our experimental objectives.

## Graphical abstract

**Figure:**
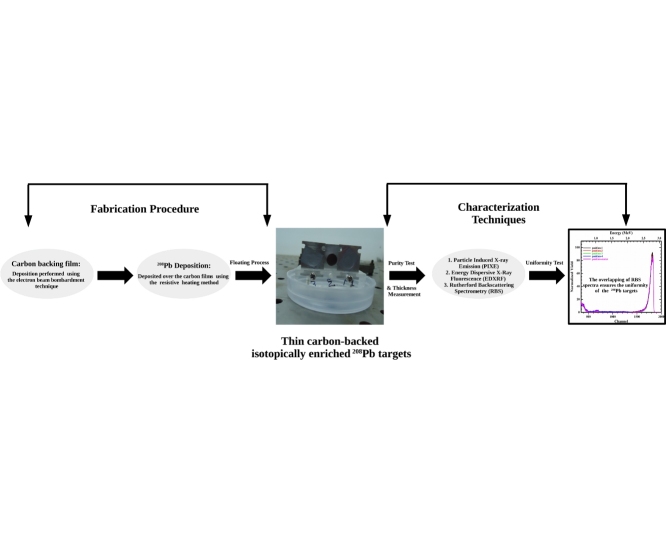

## Introduction

Thin film targets, with thickness ranging from few μg/cm^2^ to few hundreds of μg/cm^2^ are widely used in accelerator based low energy nuclear physics (projectile energy <10 MeV/u) experiments. Thin targets are the primary requisites for the success of these experiments, aimed to study nuclear reaction dynamics and nuclear structure. Formation of super-heavy elements [1] is one of the important topics in nuclear physics. The optimal selection of target and projectile combination helps in escalating the formation probability of these elements. Large number of probes such as mass-energy correlation, mass distribution and mass-gated neutron multiplicity exist to study their reaction dynamics [2]. Purity and thickness of targets are very crucial for these studies due to their strong influence on the extraction of mass and energy distribution of fission fragments. Such measurements can be done by using thin targets, either self supporting ones or backed by thin supporting materials such as carbon, gold etc. Isotopically enriched thin ^208^Pb targets with high uniformity were required for our proposed experiment to study the reaction dynamics of fusion-fission and quasi-fission processes. Use of thin targets for this experiment ensures minimum energy loss and straggling of the projectile and the reaction products inside the target. Impurities present in the target might contribute to spurious reactions with inclusion of arbitrary energy losses and consequently leading to large uncertainties in the determined masses of the fission fragments.

Different fabrication techniques such as physical vapor deposition (PVD), chemical vapor deposition (CVD) and rolling (mechanical process) [3] are available to prepare the foil targets. Each of these techniques has their specific merits and disadvantages depending upon the availability, properties and desired thickness of the target material to be fabricated. Electroplating [4], rolling [5], [6] and centrifugal precipitation [7], [8] techniques are frequently used for the preparation of thick nuclear targets (typical thickness of few mg/cm^2^). Various PVD techniques viz. ion beam sputtering, ion plating and vacuum evaporation, are being routinely used for fabrication of thin nuclear targets.

Vacuum evaporation is the best suited technique for the fabrication of uniform and thin ^208^Pb targets due to the soft and brittle nature of Pb. Fabrication of thick isotopic self-supporting Pb targets has already been reported in the literature [9], [10]. Fabrication of thick ^208^Pb sandwich targets (0.7–1.0 mg/cm^2^) using resistive heating technique has been documented by D. Marx et al. [11]. On the other hand, reports on the fabrication of thin ^208^Pb targets are rather scarce.

For our experimental measurements, the required thickness of the ^208^Pb target was ∼150 μg/cm^2^ considering minimal energy loss and beam straggling inside the target. Such a target can withstand a beam intensity of ∼1 pnA during the experiment while maintaining adequate count rate of the fission fragments produced and good spectroscopic resolution. In general, self-supporting thin targets are preferred in nuclear reaction experiments to circumvent the energy loss of heavy projectile or reaction products within the extraneous support material. However, our unsuccessful trial runs for fabrication of self-supporting targets of desired thickness led us to opt for a thin backing foil to support the ^208^Pb targets. Hence, thin enriched isotopic ^208^Pb targets have been fabricated on carbon backing in high vacuum environment at the Target Development Laboratory of Inter University Accelerator Centre (IUAC), New Delhi. In this article, we report the procedures followed to fabricate and characterize the carbon-backed thin and uniform ^208^Pb targets.

## Experimental setup

**Fig. 1:**
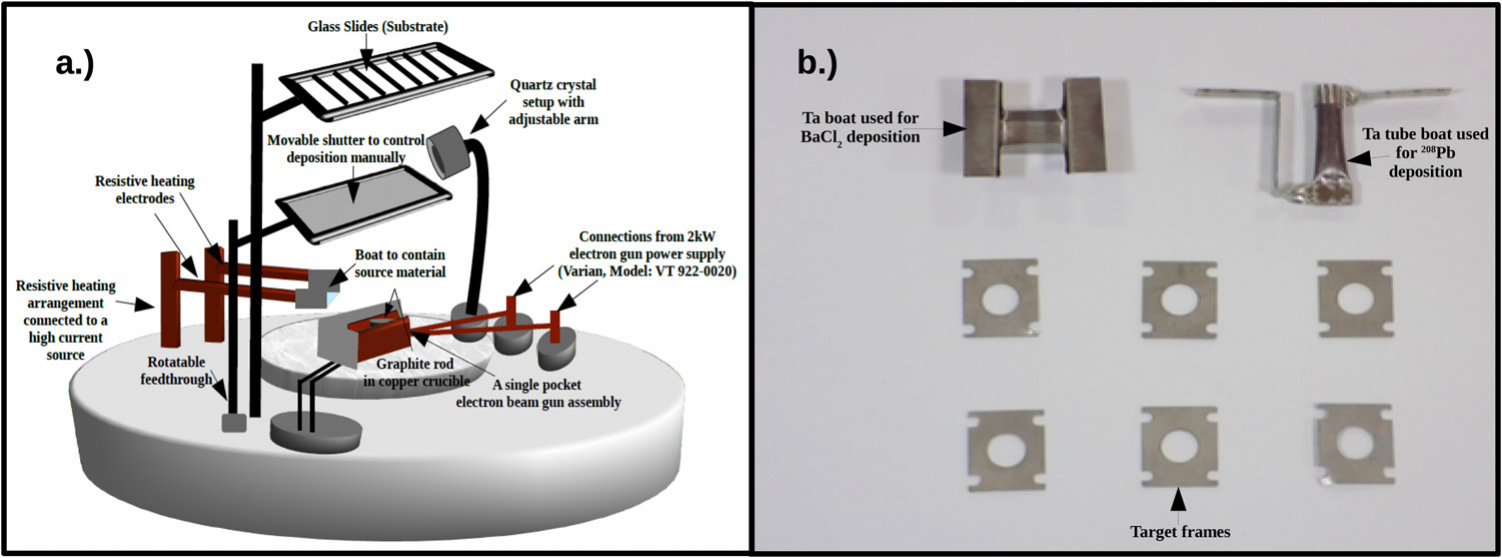
a.) Schematic diagram depicting the arrangement of various components inside the high vacuum evaporator; b.) Equipment used for the target preparation.

^208^Pb targets were fabricated on the backing of carbon foils using vacuum evaporation method. Diffusion pump based vacuum coating unit (High Vacuum Evaporator) having a resistive heating arrangement and a single pocket electron beam gun (Varian Model VT 922-0020, maximum power dissipation of 2 kW) was used for the deposition of ^208^Pb and carbon respectively. A quartz crystal thickness monitor was used to monitor and control the rate of deposition and thickness of films during the evaporation process. The details of the high vacuum evaporator are described elsewhere [10]. Fig. 1 illustrates the arrangements inside the evaporator and the equipment used for the target fabrication.

### Preparation of backing foil

Low atomic number (Z) and low Q-value makes carbon an excellent choice for backing material as it interferes minimally with the experimental measurements. In our case, carbon backing also provides an extra benefit of minimizing the fast oxidation and beam deterioration of ^208^Pb targets. Due to high melting point of carbon (3550 °C), electron beam bombardment technique was used for its deposition. For the separation of carbon from substrate (glass slide), BaCl_2_ was used as parting agent because of its ability to withstand heat generated during carbon deposition.

The first step towards the target fabrication was deposition of BaCl_2_ on clean glass slides using the resistive heating technique. A thin pellet of BaCl_2_ was made out of its powder using a hydraulic press. Afterwards, a BaCl_2_ pellet was kept in a Tantalum (Ta) boat at a distance of 19 cm from the glass slides. Initially, BaCl_2_ was deposited over the closed shutter positioned in between the source and substrate to remove undesirable contaminations from the pellet. Then, the shutter was opened and the current across the Ta boat was gradually increased to 165 A (voltage = 1 V) to deposit 100 nm of BaCl_2_ at a uniform deposition rate of 0.1 nm/s. A vacuum of 2.5 × 10^−6^ mbar was achieved and maintained during the whole evaporation process. After the successful deposition of BaCl_2_, the chamber was allowed to cool down to room temperature. Then, evaporation of carbon was performed on BaCl_2_ coated films without disturbing the vacuum inside the chamber. For this, a small piece of cylindrical graphite rod (diameter = 9 mm, length = 6 mm) which had already been placed in the copper crucible of the electron gun assembly was used. A part of the carbon surface was evaporated away and deposited over the closed shutter to eliminate unwanted contaminants and any trace metal impurities incorporated during the cutting of graphite rods. Eventually, carbon was deposited over BaCl_2_ coated films at the current and voltage of 130 mA and 220 V respectively. Using the quartz crystal monitor, the thickness of the carbon films deposited was found to be ∼ 20 μg/cm^2^. Due to development of internal stress, the carbon coated films ruptured while floating them off from the glass slides. To avoid this and to keep the carbon films stress free, the carbon coated slides were annealed at 325 °C by keeping them horizontally in a tubular furnace (Thermolyne tube furnace; Model 79400, 50 mm diameter quartz tube) for an hour in an argon environment (pressure of 2 bar). The temperature was measured by the type K (Chromel/Alumel) thermocouple arrangement within the furnace. During the whole annealing process, continuous argon gas flow was maintained at a rate of 400–500 cm^3^/min inside the tube furnace.

### Preparation of ^208^Pb targets

In view of the low melting point of Pb (327 °C), resistive heating technique was used for ^208^Pb deposition. Due to the limited availability and high cost of the isotopically enriched material (63 mg; 99.4% enrichment), trial depositions were carried out with natural Pb material prior to the fabrication of enriched isotopic targets. The fabrication technique was optimized by tuning various parameters like the current, voltage and the source to substrate distance, to ensure uniform deposition of the target material and minimal substrate degradation from heat of the source. In our trial runs, it was observed that at the minimum possible source to substrate distance (6 cm), the maximum amount of deposition occurred with minimum Pb material consumption, but the degradation of the deposited films was observed. On the other hand, with increased source to substrate distance (>19 cm), more uniformity was achieved in the films at the cost of more material consumption. Finally, deposition was carried out at an optimized source to substrate distance of 13.5 cm. The material consumption was also reduced by choosing a suitable boat for containing the target material [12]. A Ta tube boat (length = 3.4 cm, diameter of mouth = 0.6 cm) having large length to diameter ratio and small solid angle coverage was used. Target material contained in a clean Ta tube boat clamped to the electrodes was then heated by using a high current source. Current was increased until a constant deposition rate of 0.1 nm/s was achieved. In order to generate sufficient amount of heat to evaporate Pb, current was kept at 117 A during the evaporation process. Finally, all these optimized parameters and settings were adopted for the isotopic ^208^Pb deposition.

Isotopic ^208^Pb was deposited on the annealed carbon coated slides placed at the optimized distance from the source (^208^Pb) in the Ta tube boat. During the deposition, the initial current was kept at a lower value of 30 mA and then current was steadily increased to the optimized value of 117 A and the corresponding voltage was 1 V. Final deposition was made at a deposition rate of 0.1 nm/s. A vacuum of 3.4 × 10^−6^ mbar was achieved and maintained during the evaporation process. To achieve the desired thickness of targets (∼150 μg/cm^2^), 48 mg of ^208^Pb material was consumed during the evaporation process. Once the chamber had cooled down to room temperature, the slides were taken out of the chamber after venting it off with dry nitrogen. Further, to separate the carbon-backed ^208^Pb films from BaCl_2_ coated glass slides, the slides were gently immersed into warm distilled water at an inclination of ∼45° with respect to the water surface. During this floating process, special care was taken to ensure the minimum direct contact of the target films with water. Afterwards, the target frames were slowly brought under the floating foils and the foils were carefully fished onto the central aperture (10 mm diameter) of the stainless steel target frames (20 mm × 25 mm). As the foils were quite thin (total thickness ∼220 nm), they naturally adhere to the peripheral region of the stainless steel frames. This adhesion was sufficiently strong such that it was not possible to separate these foils from the frames without destroying them.

## Thickness measurement and characterization of targets

Different techniques employed to ensure the purity and adequate thicknesses of the targets are described as follows:

### Alpha particle transmission method

Alpha particle transmission method was used to measure the thickness of targets by estimating the energy loss of 5.486 MeV alpha particles emitted from ^241^Am source in the target foil. As ^208^Pb targets have backing of carbon, so our first step was to estimate the exact energy loss of alpha particles inside the carbon. Two sets of carbon films were fabricated under identical conditions. Foils from one of the sets were floated and used for thickness measurement while the slides from the other set were used for deposition of ^208^Pb films. The energy loss inside the carbon foil was measured by taking the difference in the peak centroids of the measured energy spectra of the alpha particles with carbon foil and blank target frame (without any foil). In the same way, energy loss of alpha particle was determined for the carbon-backed ^208^Pb target. Then energy loss for ^208^Pb was calculated after correcting for the energy loss due to carbon. Finally thickness of the target was evaluated using this energy loss and stopping power (extracted from SRIM code [13]) of alpha particles in a ^208^Pb target. The thicknesses of the ^208^Pb targets were found to be lying between 60 μg/cm^2^ and 250 μg/cm^2^. In this measurement, an uncertainty of ∼20% was observed considering the errors in the source-foil-detector geometry, error in the evaluation of peak centroid and due to the uncertainty in the stopping power calculation. For one of the targets having a thickness of 115 ± 23 μg/cm^2^, further characterization techniques were employed to confirm its thickness and purity.

### Particle Induced X-ray Emission (PIXE)

To perform elemental analysis of ^208^Pb target, Particle Induced X-ray Emission (PIXE) measurements [14] have been carried out at the Cyclotron Laboratory of Panjab University, Chandigarh. A ^208^Pb target was positioned perpendicularly to the beam axis and irradiated with 2.7 MeV proton beam. Characteristic X-rays of elements were detected by using the Canberra HPGe detector (model GUL0035) positioned at a backward angle of 45° with respect to the beam direction.

**Fig. 2:**
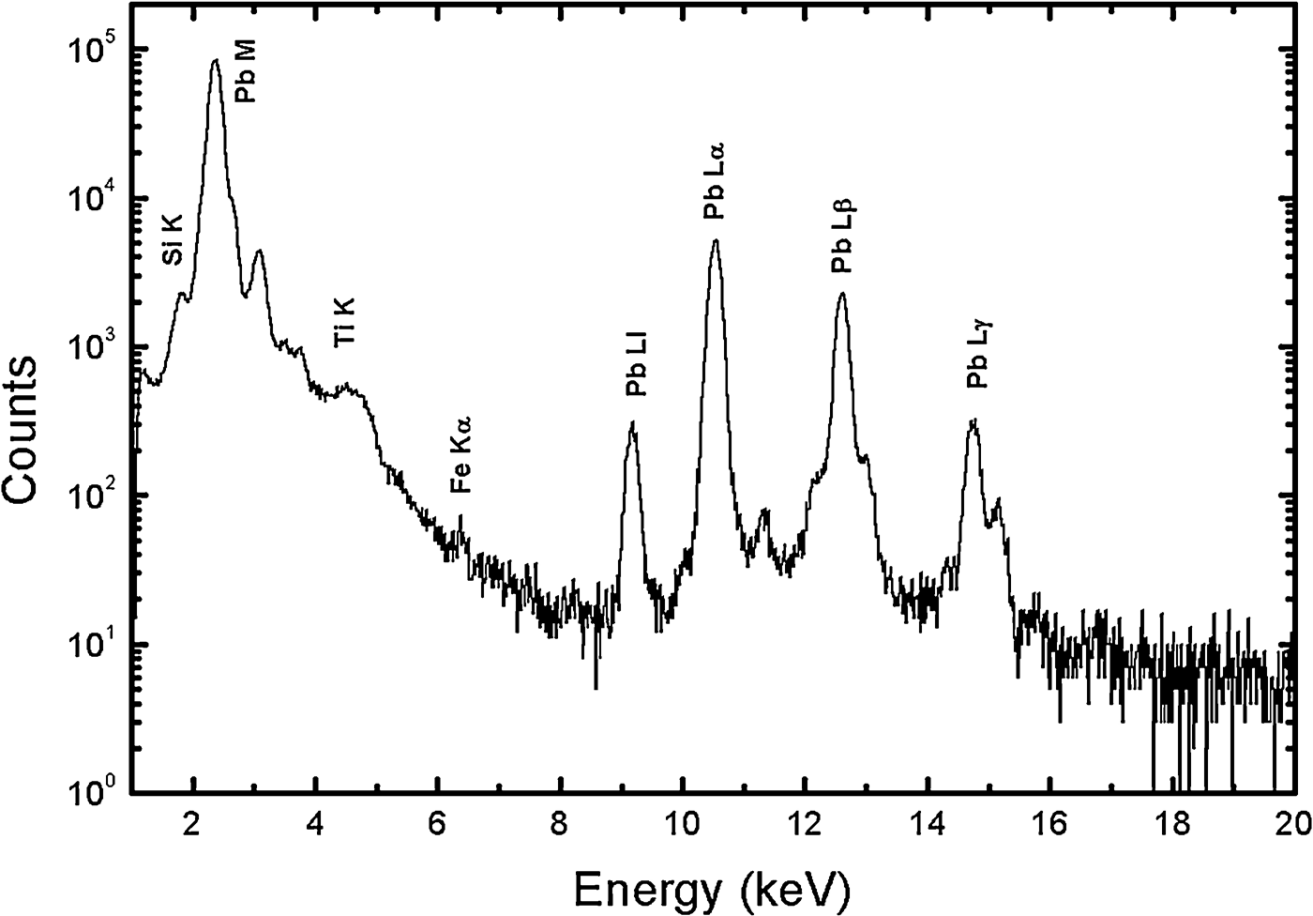
PIXE spectrum of the ^208^Pb target.

Fig. 2 shows the PIXE spectrum acquired for the ^208^Pb target. Here, in addition to characteristic *L* and *M* X-rays corresponding to ^208^Pb, characteristic X-rays for elements *Fe*, *Ti* and *Si* were also observed. The data analysis was performed using the GUPIX software package [15] and the extracted thicknesses of the elements are tabulated in Table 1. The thicknesses of the detected impurities were found to be less than 1 μg/cm^2^. Moreover, these extents of impurities are not expected to interfere in our measurements with heavy projectiles (^48^Ti) at an incident energy of 275 MeV. The detection limit of the PIXE experiment is of the order of 1 ng/cm^2^. Hence, it can be ascertained that apart from the observed impurities, no other impurities at concentrations prohibitive to the experiment are present in the target. The thickness of ^208^Pb target was found to be 109 ± 10 μg/cm^2^, consistent with the value obtained using the alpha particle transmission method.

### Energy Dispersive X-Ray Fluorescence (EDXRF)

To check the impurities incorporated during the fabrication process of the targets, Energy Dispersive X-Ray Fluorescence (EDXRF) technique has been employed at EDXRF laboratory of Department of Physics, Panjab University, Chandigarh. The reflection mode geometrical arrangement having the ^241^Am annular source, Si(Li) detector and Al-lined Pb collimator was used to analyze ^208^Pb targets. Targets were bombarded with 59.54 keV γ-rays emitted from the ^241^Am source.

**Fig. 3:**
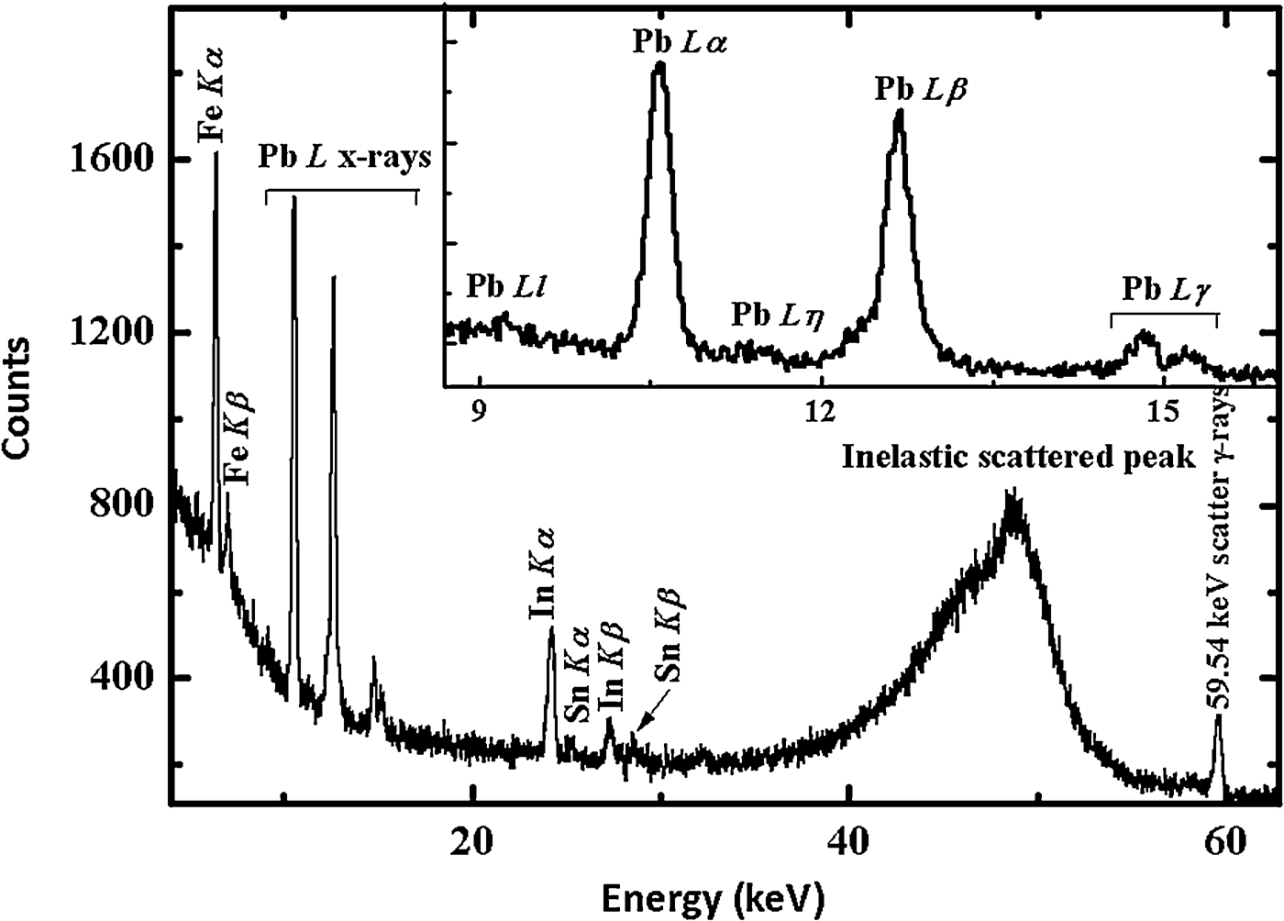
Typical spectrum of the ^208^Pb target obtained using EDXRF setup.

The full X-rays characteristic spectrum for ^208^Pb target from EDXRF setup [16] is shown in Fig. 3. The inset of Fig. 3 depicts the *L* X-rays of ^208^Pb. The spectrum also shows the *K* X-rays of *Fe*, *In* and *Sn* along with *L* X-rays from ^208^Pb target. *L* X-rays peak of *In* emerged from the Si(Li) crystal in the detector. *Fe* and *Sn* peaks were originated from the iron collimator and *Sn* shielding used in the geometrical setup respectively. Due to insensitivity of EDXRF technique for materials having atomic number less than 13, characteristic lines corresponding to carbon backing were not observed. Apart from characteristic X-rays lines corresponding to EDXRF geometrical setup and ^208^Pb, no other X-rays lines coming from any other element were observed. Hence, the purity of targets is strongly revealed through this analysis. The target thickness was also cross-checked using EDXRF technique. Here, the basic parameter technique has been used to determine the thickness of target. The net area count after the background corrections in each photo peak was used in the calculation of thickness of the target. For this ^208^Pb target, the thickness was found to be 106 ± 14 μg/cm^2^, which matches well with the values obtained using alpha transmission method and PIXE technique.

### Rutherford Backscattering Spectrometry (RBS)

**Fig. 4:**
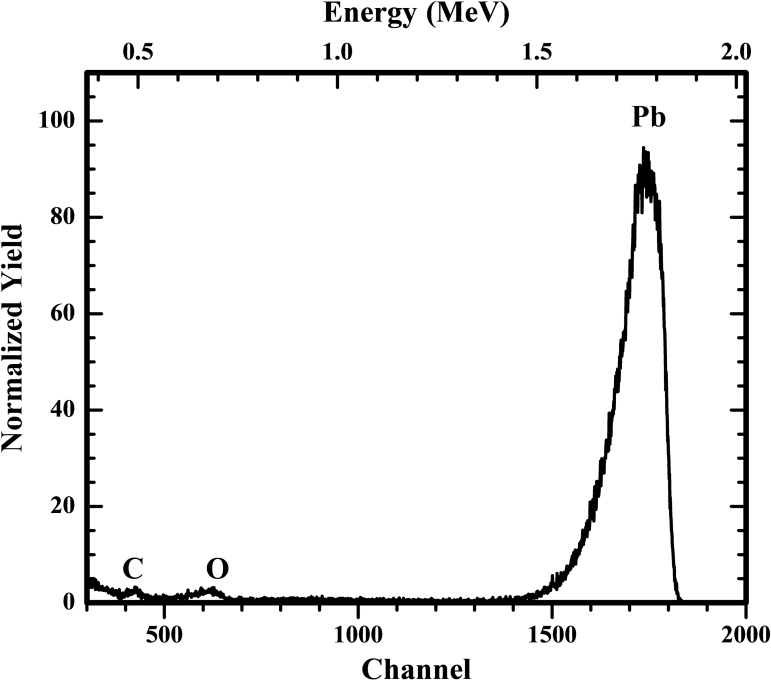
Experimental RBS spectrum of the ^208^Pb target.

In view of oxidation characteristics of Pb, targets need to be scrutinized for their oxygen contents. In order to determine the oxygen contents and to check the presence of impurities having atomic number less than 13, the region in which PIXE and EDXRF techniques are insensitive, Rutherford Backscattering Spectrometry (RBS) measurement was performed for ^208^Pb target using Pelletron Accelerator RBS-AMS Systems (PARAS) at IUAC. Data analysis of experimental spectra of 2 MeV He backscattered from carbon-backed ^208^Pb targets at scattering angle of 165° was performed using RUMP [17] and SIMNRA [18] software packages. Oxygen contents were found within 10% in number density in ^208^Pb target which was acceptable for our experiments. Fig. 4 shows the experimental RBS spectrum for ^208^Pb target having three peaks marked as carbon (C), oxygen (O) and lead (Pb). In addition to carbon backing on the target, one carbon block was also kept behind the foil to avoid backscattering of alpha particles from other materials like stainless steel. Hence the number density of carbon could not be determined in this RBS measurement. Thickness of this target was found to be 110 ± 6 μg/cm^2^ which is in accordance with the values calculated using PIXE, EDXRF and alpha transmission method.

**Fig. 5:**
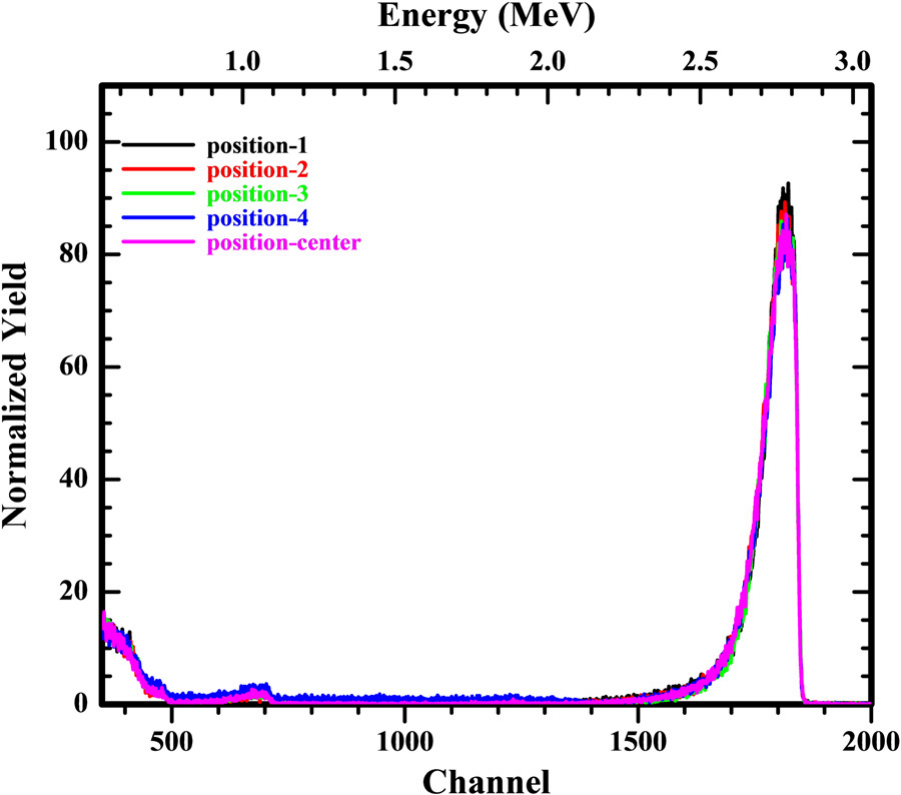
Experimental RBS spectra of the ^208^Pb target, here different line colors correspond to different mapped positions on the target.

To check the uniformity of the target, RBS measurements were also performed at center and four evenly spaced (2 mm from the center) positions marked around the center of the target using a collimated beam spot of 1 mm in diameter. The results show that the target was highly uniform as the maximum variation in thickness at all the mapped positions is less than 1% of the total thickness which can also be observed from the seamless overlapping of all the five RBS spectra (Fig. 5).

## Experimental results

**Fig. 6:**
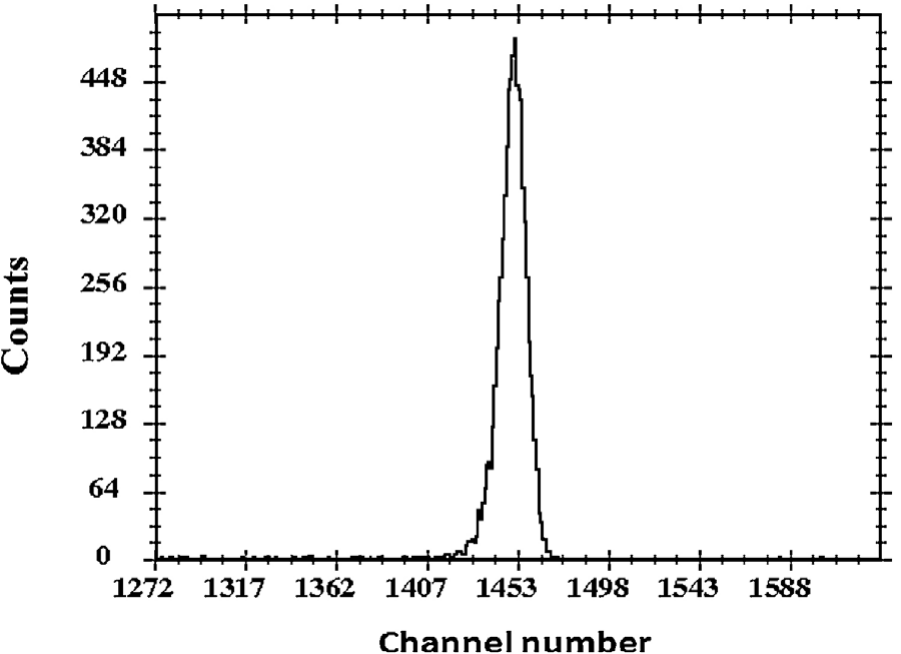
Energy spectrum of the ^48^Ti scattered from the ^208^Pb target.

Thin and uniform ^208^Pb targets fabricated using the above process were successfully used in the experiments carried out using the Pelletron + LINAC and NAND [19] facility at IUAC. A pulsed beam of ^48^Ti (E_lab_ = 275 MeV, intensity = 0.7 pnA) was bombarded on thin ^208^Pb target to populate near super-heavy nucleus ^256^Rf. At this energy, the projectile suffered an energy loss of 1.68 MeV inside the target. Our main aim is to disentangle the contribution of fusion-fission and quasi-fission processes in near super-heavy nucleus ^256^Rf using mass-energy correlation and mass-gated neutron multiplicity as a probe. In this experiment, neutrons were detected using an array of 100 neutron detectors (organic scintillator: BC501 A) of the NAND facility, in coincidence with the fission fragments detected using large area multi-wire proportional counter (MWPC). Beam was monitored using two Si surface barrier detectors (SSBD) kept at an angle of ±13.5° with respect to the beam axis. Fig. 6 shows the energy spectrum (energy calibration factor of 189 keV/channel) of elastically scattered particles from one of the SSBD. Energy resolution was found to be 800 keV which is acceptable for such experiments. From the analysis of experimental data, mass resolution of fission fragments was found to be ∼10 a.m.u. which clearly reveals that for such a heavy system, there was minimal energy straggling inside the ^208^Pb target. Moreover, with a typical beam intensity of 0.7 pnA the energy deposition rate in this target was 0.46 mW. Despite this, the target integrity was intact throughout the whole experiment spanning 240 h of continuous beam bombardment. Furthermore, RBS analysis of the target after the experiment showed no variation in its thickness nor in its uniformity owing to the conducting nature of the carbon backing film.

## Conclusions

Thin and uniform ^208^Pb targets having thickness ranging from 60 μg/cm^2^ to 250 μg/cm^2^ with carbon backing of thickness ∼20 μg/cm^2^ were successfully prepared by the vacuum evaporation method. Purity and thickness of these targets were analyzed by PIXE, EDXRF and RBS techniques, which ensured the absence of any considerable impurity in the targets. Thickness of targets measured using the different characterization techniques were found to be in good agreement with each other. Uniformity of targets was also verified using RBS technique. Precise measurement of the target thickness helped in extracting the mass-energy distribution of fission fragments produced in ^48^Ti + ^208^Pb reaction by determining accurate energy loss of the fission fragments inside the target.

## Figures and Tables

**Table 1:** Thickness of elements detected through PIXE analysis.

Element	Thickness (μg/cm^2^)
Pb	109.00 ± 10.00
Fe	0.021 ± 0.001
Ti	0.071 ± 0.004
Si	1.03 ± 0.10
